# Treatment of Bleeding Gastric Varices by Endoscopic Cyanoacrylate Injection: A Developing-country Perspective

**DOI:** 10.7759/cureus.7062

**Published:** 2020-02-20

**Authors:** Muhammad Mansoor-Ul-Haq, Abdul Latif, Mansoor Asad, Farooque Aziz Memon

**Affiliations:** 1 Gastroenterology, Liaquat National Hospital and Medical College, Karachi, PAK; 2 Gastroenterology, Avicenna Medical College, Lahore, PAK; 3 Gastroenterology, Jinnah Postgraduate Medical Centre, Karachi, PAK

**Keywords:** gastric varices, bleeding gastric varices, endoscopy, cyanoacrylate

## Abstract

Introduction

Gastric varices (GV) are less commonly seen but bleed more severely than esophageal varices (EV). Transjugular intrahepatic portosystemic shunt (TIPS), alcohol injection, and N-butyl-2-cyanoacrylate (NBCA) are generally used for GV bleed management. NBCA is usually injected endoscopically and is known to be quite successful in the treatment of GV bleeding. This study was conducted with the objective of determining the outcomes in patients with GV treated with NBCA.

Methods

We conducted a retrospective review of medical records and reports of endoscopy performed between March 2015 and June 2018 at a large tertiary care center. Patients of any age and gender having a history of chronic liver disease, those presenting with hematemesis or melena, and those who were found to have GV bleed on endoscopy and treated with NBCA were included. All the endoscopy procedures were undertaken within 24 hours of admission. Informed written consent was obtained from all the patients before the procedure. The outcomes were measured in terms of rate of mortality, hemostasis achieved, duration of stay at the hospital, and requirement of blood transfusion.

Results

A total of 31 patients met the inclusion criteria; (58.1%) were males and 13 (41.9%) were females. The mean age was 55.23 ±8.77 years; 12.9% were Child-Pugh class-A, 64.5% were class B, and 22.6% were class C. Out of the 31 patients, 27 (87%) patients achieved hemostasis. Moreover, 22 (71%) patients had hospital stay ranging between 5-8 days. The overall mortality rate was 9.7% (3 patients). No complication was reported from NBCA injection.

Conclusion

The injection of NBCA can provide a safe and effective method for the management of GV bleeding as demonstrated by the results of our study, which showed hemostasis in a majority of the cases after the initial injection with no procedure-related complications, a reduced hospital stay, and a reduced rate of mortality.

## Introduction

Patients suffering from chronic liver disease may develop collateral circulation in the form of varices. These are commonly seen in distal one- third of the esophagus, the stomach, perigastric, perisplenic, and peripancreatic regions. In some cases, they may be seen in and around the rectum and also along the anterior abdominal wall. Varices within the stomach, also termed as gastric varices (GV), are seen less commonly. However, bleeding from GV is quite lethal. Its management is challenging, requiring a high amount of blood transfusion, as it can cause an increased rate of rebleeding and increased mortality risk [1]. GV bleeding can be managed by various means; one option is the injection of N-butyl-2-cyanoacrylate (NBCA), or simply cyanoacrylate, under endoscopic guidance [2]. Transjugular intrahepatic portosystemic shunt (TIPS) and balloon-occluded retrograde transvenous obliteration (BRTO) may also be used for the management of GV bleeding [2].

Many patients with GV may also have portal hypertension. Thrombosis of the splenic vein is not commonly seen in such patients [3]. GV is classified as primary when it is accompanied by esophageal varices (EV), whereas secondary GV occurs after the obliteration of EV [4]. The prevalence of GV is approximately 15% and the risk of bleeding from GV is approximately 22.7% [5].

Sometimes, the endoscopic treatment of hemorrhagic varices fails to stop the bleeding. Such cases require surgical management. New agents have been recently developed for management by endoscopic means and by the assistance of interventional radiology for improving the GV bleed outcome [3]. However, no consensus has been achieved regarding the optimal management of GV. The treatment of GV with cyanoacrylate has shown good results, with a high hemostasis rate and reduced early and late rebleeding rates as compared to other endoscopic modalities [6]. Endoscopic cyanoacrylate injection for GV is practiced in many countries. However, cyanoacrylate has not gained universal acceptance. We report the use of cyanoacrylate for GV bleeding management from a developing-country perspective. The main objective of this study was to determine the outcome in patients with GV bleeding treated by cyanoacrylate.

## Materials and methods

We conducted a retrospective review of medical records and reports of endoscopy performed between March 2015 and June 2018 at a large tertiary care center. Patients with GV bleeding were identified by reviewing well-maintained medical records and endoscopy reports. Demographic and clinical characteristics of the patients such as age, gender, endoscopic procedures performed, their findings, and clinical outcomes in terms of hemostasis, mortality, procedure-related complications, and length of hospital stay were recorded. Hemostasis was labeled as achieved if the vital signs remained stable after the procedure without any evidence of rebleeding within 24 hours. Procedure-related complications such as fever, any local ulceration, or any organ embolization were noted. Patients of any age and gender, having a history of chronic liver disease (CLD), presenting with hematemesis or melena of any duration, and those who had undergone upper GI endoscopy and were found to have GV bleeding were included. Patients with hepatic encephalopathy or those having non-cirrhotic portal hypertension were excluded. Endoscopy was performed within 24 hours of admission after taking informed written consent from the patients. Before the procedure, intravenous octreotide was initiated in all cases. On endoscopy, active spurter or clotted dark brown mass of blood in the fundus of stomach was labeled as GV bleeding. For the endoscopic injection of NBCA, the Histoacryl (B. Braun, Melsungen, Germany) glue working solution was prepared; 0.8 ml of lipiodol was mixed with 0.5 ml Histoacryl glue. A large-bore sclerotherapy needle was primed with a small amount (approximately 0.8-1.2 ml) of lipiodol. This was done to make sure that Histoacryl glue did not solidify prematurely. Under endoscopic guidance, bleeding vessel was identified and punctured followed by the solution injection into it. Sterile distilled water injection (up to 1-2 ml) was immediately followed to make sure that the entire volume of the solution had been instilled into the gastric varix. Following injection, to maintain needle patency, it was immediately flushed with distilled water. This ensured that the needle remained patent. The medical records of follow-up were reviewed to see the post-procedure outcome. Data analysis was performed with the Statistical Package for Social Sciences (SPSS) software version 18 (IBM, Armonk, NY). Mean with standard deviation (SD) was calculated for quantitative variables. The frequency with percentage was calculated for qualitative variables of outcomes such as hemostasis, mortality, and requirement of blood transfusion.

## Results

A total of 31 cases of GV bleeding treated with NBCA injection were identified from the medical records and included in the study. The mean age of the patients was 55.23 ±8.77 years (range: 38-72 years). The majority of the patients were male (n = 18, 58.1%). All cases of GV bleeding were found to be caused by cirrhosis related to hepatitis B virus or hepatitis C virus infection. According to Child-Pugh classification, 12.9% had class A liver status, 64.5% had class B liver status, and 22.6% had class C liver status respectively. Baseline characteristics are summarized in Table 1.

**Table 1:** Baseline characteristics of the patients ǂmean ±standard deviation

Baseline characteristics (n = 31)		
	Number	Percent
Age, years	55.23 ±8.77^ǂ^	
Gender		
Males	18	58.1
Females	13	41.9
Clinical presentation		
Hematemesis	24	77.4
Melena	7	22.6
Etiology of cirrhosis		
Hepatitis B	5	16.2
Hepatitis C	26	83.8
Child-Pugh classification		
Class A	4	13
Class B	20	64.5
Class C	7	22.5

Hemostasis was achieved in 27 (87%) cases with a single session of NBCA injection. Four patients did not achieve hemostasis. Out of those four patients, one was referred for the TIPS procedure. Among the total 31 patients, mortality was observed in 3 (9.7%). The causes of death included advanced cirrhosis and rebleeding of GV.

Among patients requiring transfusion before the endoscopy or during their hospital stay, 54.8 % required ≤3 pints of blood, 32.3% required 4-6 pints of blood, and 12.9% required ≥7 pints of blood; 22 patients (71.0%) had a hospital stay of 5-8 days. No procedure-related complications were noted.

## Discussion

GV can develop in patients suffering from portal hypertension and may account for upper gastrointestinal bleed. GV can also be seen in patients with extrahepatic portal vein obstruction occurring mainly due to pancreatitis or in association with pancreatic neoplasm. In the current study, we determined the outcomes of NBCA injection in patients treated for GV bleeding. The treatment options available for GV bleeding are not well established; however, endoscopic injection of NBCA is a widely recommended option in literature [7,8]. The best alternatives to endoscopic treatment are TIPS and BRTO. The treatment of patients undergoing BRTO is usually complicated and requires a team approach.

In our study, 31 known patients of chronic liver disease who had presented with upper GI bleed were included. Males were higher in number in our study cohort than females. Male predominance was also found in another international study [9]. In our study, 58.1% of the patients were males. We reported a slightly lower mean age as compared to the mean age reported by Mosli et al. [9].

In our study, the endoscopic injection of NBCA was found to be quite effective for the management of GV bleeding. After the initial injection, hemostasis was achieved in almost 87% of the patients in our study. In a previous study by Mosli et al, the rate of hemostasis achieved was approximately 79% [9]. However, in another international study, the initial hemostasis was achieved in 98.4% of the cases, which was higher than the rate reported in our study [7]. According to a study of associated diagnosis of hepatocellular carcinoma (HCC), a high model for end-stage liver disease (MELD) score, advanced stage of the tumor, and increased level of alanine transaminase are poor prognostic factors and associated with a higher risk and both short- and long-term mortality [10].

According to another international study, endoscopic NBCA injection resulted in a high rate of hemostasis of approximately 95% in the treatment of GV bleeding [11]. Rebleeding was evident in 8.1% of the cases [11]. In another study, hemostasis was reported in 97.8% of the patients, which is higher than the rate reported in our study [12]. The overall mortality reported in our study was 9.7%, which was lower than the one reported in the Canadian population in a study [11]. Another study reported a higher mortality rate of 21.1% as compared to the one reported in our study [12].

According to the results of a randomized controlled trial that was conducted in patients having isolated fundal varices, which used cyanoacrylate versus alcohol injection, hemostasis rate was higher with cyanoacrylate injection as compared to alcohol injection [13]. Cyanoacrylate glue injection achieved hemostasis in 89% of cases whereas hemostasis was achieved in 62% of the cases with alcohol. Moreover, cyanoacrylate was more effective in achieving obliteration as compared to alcohol [13]. According to the results of another prospective randomized trial comparing NBCA injection with band ligation, NBCA had a higher initial rate of hemostasis and a lower rate of rebleeding as compared to band ligation [14].

Another randomized controlled trial comparing TIPS and NBCA demonstrated that variceal obliteration was higher in the NBCA group as compared to the TIPS group. However, the study also demonstrated that TIPS was more effective in the prevention of rebleeding [15].

No procedure-related complication was reported in our study and our results are comparable to the one reported by another author [16]. However, procedure-related complications can occur after the endoscopic injection of NBCA. Cheng et al. reported sepsis in 1.3% of patients and distant embolism in 0.7% of the patients [17]. Systemic embolization is a serious side effect of NBCA injection. According to a study, NBCA glue can have embolic side effects such as stroke as a result of thromboembolism in the anterior or posterior circulation, or pulmonary embolism (PE), splenic embolism, or portal vein thrombosis [18]. It may also cause multi-organ infarction in the presence of patent foramen ovale or the presence of any arteriovenous (AV) pulmonary shunt [18]. NBCA glue may also act as a septic focus resulting in recurrent sepsis [18].

Our study had some limitations. This was a retrospective study conducted with a small sample size. However, despite this limitation, we believe that our results are almost comparable to the ones reported by other authors. It is recommended that further studies should be carried out in multiple centers incorporating variables such as late rebleeding, transfusion of fresh frozen plasma (FFP), and level of total bilirubin so that their effects on prognosis could also be evaluated.

## Conclusions

GV bleeding represents a grave complication of chronic liver disease. Our study results demonstrate that the injection of NBCA via endoscopic guidance is safe and effective in the management of GV bleeding. The majority of our patients achieved hemostasis after the initial injection with a reduced length of hospital stay and low mortality. No procedure-related complications were observed.

## References

[1] Sarin SK, Mishra SR (2010). Endoscopic therapy for gastric varices. Clin Liver Dis.

[2] Goral V, Yılmaz N (2019). Current approaches to the treatment of gastric varices: glue, coil application, TIPS, and BRTO. Medicina (Kaunas).

[3] Saad WE, Darcy MD (2011). Transjugular intrahepatic portosystemic shunt (TIPS) versus balloon-occluded retrograde transvenous obliteration (BRTO) for the management of gastric varices. Semin Intervent Radiol.

[4] Ryan BM, Stockbrugger RW, Ryan JM (2004). A pathophysiologic, gastroenterologic, and radiologic approach to the management of gastric varices. Gastroenterology.

[5] Mumtaz K, Majid S, Shah H, Hameed K, Ahmed A, Hamid S, Jafri W (2007). Prevalence of gastric varices and results of sclerotherapy with N-butyl 2 cyanoacrylate for controlling acute gastric variceal bleeding. World J Gastroenterol.

[6] Lim YS (2012). Practical approach to endoscopic management for bleeding gastric varices. Korean J Radiol.

[7] Kang EJ, Jeong SW, Jang JY (2011). Long-term result of endoscopic Histoacryl®(N-butyl-2-cyanoacrylate) injection for treatment of gastric varices. World J Gastroenterol.

[8] Kozieł S, Kobryń K, Paluszkiewicz R, Krawczyk M, Wróblewski T (2015). Endoscopic treatment of gastric varices bleeding with the use of n-butyl-2 cyanoacrylate. Prz Gastroenterol.

[9] Mosli MH, Aljudaibi B, Almadi M, Marotta P (2013). The safety and efficacy of gastric fundal variceal obliteration using N-butyl-2-cyanoacrylate; the experience of a single Canadian tertiary care centre. Saudi J Gastroenterol.

[10] Chang CJ, Hou MC, Liao WC, Lee FY, Lin HC, Lee SD (2012). Risk factors of early re-bleeding and mortality in patients with ruptured gastric varices and concomitant hepatocellular carcinoma. J Gastroenterol.

[11] Al-Ali J, Pawlowska M, Coss A, Svarta S, Byrne M, Enns R (2010). Endoscopic management of gastric variceal bleeding with cyanoacrylate glue injection: safety and efficacy in a Canadian population. Can J Gastroenterol.

[12] Prachayakul V, Aswakul P, Chantarojanasiri T, Leelakusolvong S (2013). Factors influencing clinical outcomes of Histoacryl® glue injection-treated gastric variceal hemorrhage. World J Gastroenterol.

[13] Sarin SK, Jain AK, Jain M, Gupta R (2002). A randomized controlled trial of cyanoacrylate versus alcohol injection in patients with isolated fundic varices. Am J Gastroenterol.

[14] Lo GH, Lai KH, Cheng JS, Chen MH, Chiang HT (2001). A prospective, randomized trial of butyl cyanoacrylate injection versus band ligation in the management of bleeding gastric varices. Hepatology.

[15] Lo GH, Liang HL, Chen WC (2007). A prospective, randomized controlled trial of transjugular intrahepatic portosystemic shunt versus cyanoacrylate injection in the prevention of gastric variceal rebleeding. Endoscopy.

[16] Seewald S, Ang TL, Imazu H (2008). A standardized injection technique and regimen ensures success and safety of N-butyl-2-cyanoacrylate injection for the treatment of gastric fundal varices (with videos). Gastrointest Endosc.

[17] Cheng LF, Wang ZQ, Li CZ, Lin W, Yeo AE, Jin B (2010). Low incidence of complications from endoscopic gastric variceal obturation with butyl cyanoacrylate. Clin Gastroenterol Hepatol.

[18] Kobilica N, Flis V, Sojar V (2012). Major complication after Histoacryl injection for endoscopic treatment of bleeding peptic ulcer. Endoscopy.

